# Editorial: Recent advances in the molecular genetics of glioma

**DOI:** 10.3389/fgene.2024.1435186

**Published:** 2024-07-17

**Authors:** Caroline Chung, Pawel Buczkowicz

**Affiliations:** ^1^ Department of Radiation Oncology, University of Texas MD Anderson Cancer Center, Houston, TX, United States; ^2^ PhenoTips, Toronto, ON, Canada

**Keywords:** molecular genetics, cancer genetics, glioma, immunotherapy, brain tumor, brain cancer, neuro-oncology

Gliomas, a varied and deadly group of central nervous system tumors of glial origin, are the leading cause of cancer-related death among females between 0 and 19 years and males between 0 and 39 years of age (Ostrom et al., 2022; Wang et al., 2022), highlighting the urgent need for effective diagnostic and therapeutic strategies. The molecular genetics of gliomas represent one of the most challenging and dynamic frontiers in modern oncology. Understanding the molecular genetics of glioma has become paramount in the pursuit of effective therapeutic strategies. Advances in molecular profiling have identified key genetic alterations driving gliomagenesis, offering new opportunities for targeted therapies. Single gene-targets, including mutations in genes such as EGFR, IDH, and TP53, have been extensively studied for their roles in glioma pathogenesis (Cheng and Guo, 2019; Khayari et al., 2022). Innovative treatments, including small molecule inhibitors, monoclonal antibodies, and gene editing technologies are being explored to selectively inhibit oncogenic pathways and disrupt tumor growth (Huang et al., 2022; Chen et al., 2024; Stitzlein et al., 2024). Precision medicine approaches targeting specific genetic aberrations hold promise for personalized treatment strategies tailored to individual glioma subtypes. This Research Topic highlights ten groundbreaking articles that delve into the complex molecular genetics of gliomas, including three key avenues of leveraging the genomic and molecular information for therapeutic interventions: single-targets, pan-targets, and immunotherapy.

Dysregulation of gene expression has long been correlated with the molecular landscape of gliomas. In this Research Topic, several manuscripts explore expression profiles of glioma and correlate them to prognosis. Zhao et al. uncovered a <1% mutation rate in *KIF23* using RNA sequencing and Whole-Exome Sequencing (WES) when screening 319 glioma samples. Subsequent gene-set enrichment analysis (GSEA) and review of copy number alterations (CNAs) deduced from WES data, the authors concluded that overexpression of *KIF23* was correlated with tumor samples having amplifications of the genetic region encompassing that gene, and the overexpression was positively correlated with WHO tumor grade and a worse overall survival. The analysis of gene expression of *ANXA1* in glioma by Zhang et al. utilizing three public data sources suggested the overexpression of this gene is associated with poor prognosis and that the elevated expression was an independent prognostic factor in glioma. Liu et al. identified elevated *ESPL1* expression in glioma using GSEA and correlated the findings to poor overall survival. The authors performed *in silico* analysis of potential drug targets using CMap, identifying antimycin A, thioguanosine, and zidovudine as potential small-molecules inhibitors of this gene’s signaling pathway. In a meta-analysis of four glioma datasets (TCGA, CGGA, GSE6011 and Rembrandt) by Zhou et al., high level expression of GAS2L3 was associated with higher grade tumor and shorter overall survival. Yang et al. found that centromeric protein A (CENP-A), a protein involved in chromosomal segregation during cell division, was upregulated in gliomas and high CENP-A levels were associated with high grade, response to therapy and shorter overall survival.

In contrast to their high-grade counterparts, low-grade glioma (LGG), which are categorized as WHO Grade I or II, pose different challenges driven by biological processes highlighted in three articles included in this Research Topic. Shi et al. conducted gene expression profiling of 433 LGG patients available in the TCGA database, identifying *JAG1* overexpression and Notch pathway activation as indicators of poorer prognosis and immune response in LGG. Implications of E3-related gene signatures in LGG was investigated by Tan *et al.* who identified *AURKA, MAP3K1, PCGF2, PRKN, TLE3, TRIM17,* and *TRIM34* as part of an E3-related prognostic signature and its role in LGG prognosis and tumor immune microenvironment cell infiltration. Lin et al. investigated six autophagy-related genes (BAG1, PTK6, EEF2, PEA15, ITGA6, and MAP1LC3C) to construct an autophagy-related prognostic risk model in LGG that was validated as an independent risk predictor for survival. These studies emphasize the crucial importance of gene expression profiles in understanding glioma prognosis and shaping therapeutic strategies.

To leverage the genomic and molecular information for therapeutic intervention, while single gene-targeted therapies have shown efficacy in subsets of glioma patients, the inherent heterogeneity within tumors often leads to therapeutic resistance and disease progression. Pan-targeted approaches aim to overcome this challenge by simultaneously inhibiting multiple signaling pathways involved in glioma progression. Multi-targeted kinase inhibitors, epigenetic modulators, and combination therapies are being developed to comprehensively disrupt tumor cell survival and proliferation. By targeting interconnected signaling networks, pan-targeted therapies offer the potential for synergistic effects and improved clinical outcomes in glioma patients. The need for combination therapy is blatantly apparent in the most aggressive and inoperable subtypes of glioma, those affecting the brainstem such as Diffuse Intrinsic Pontine Glioma (DIPG). Due to an intact blood-brain-barrier and unique molecular profile, these tumors have unique treatment challenges.

Immunotherapy has emerged as a promising approach across many solid tumors including for the treatment of gliomas. Strategies such as immune checkpoint inhibitors, chimeric antigen receptor T (CAR-T) cell therapy, and peptide vaccines are being investigated to enhance anti-tumor immune responses. By modulating immune checkpoints or directly engaging immune effector cells, immunotherapy offers a novel paradigm for glioma management, aiming to overcome immunosuppressive mechanisms within the tumor microenvironment. Lin et al. review the most recent advances in emerging technologies such as Magnetic Resonance guided Focused Ultrasound (MRgFUS) and various immunotherapy treatments such as cancer vaccines, autologous cell transfer therapy, CAR-T cell therapy, and immune checkpoint blockers for DIPG. Regarding immune related biomarkers, long noncoding RNAs (lncRNAs), which are noncoding RNAs that are more than 200 nucleotides in length without significant protein-coding function have been shown that these are immune-related and are prognostic.

In summary, the complex and heterogeneous molecular genetics of gliomas pose both daunting challenges and exciting opportunities ripe for therapeutic exploration. Immunotherapy, single gene-targets, and pan-targets represent distinct yet complementary strategies in the pursuit of more effective and personalized treatments for glioma patients. Ongoing research is imperative to unravel the intricate genetic and molecular mechanisms driving glioma development and resistance, paving the way for groundbreaking interventions that could dramatically improve patient survival, outcomes, and quality of life.
